# On the Symptoms of Epidemic Cholera

**Published:** 1833-06

**Authors:** Alexander Turnbull Christie

**Affiliations:** the Honourable East-India Company's Service; Member of the Royal Asiatic Society of Great Britain and Ireland, of the Wernerian and Royal Medical Societies of Edinburgh, &c.


### THE EPIDEMIC CHOLERA.

*On the Symptoms of Epidemic Cholera.* By Alexander Turnbull Christie, M.D., of the Honourable East-India Company's Service; Member of the Royal Asiatic Society of Great Britain and Ireland, of the Wernerian and Royal Medical Societies of Edinburgh, &c.

(Continued from p. 365.)

The epidemic cholera does not always present exactly the same symptoms, and almost every visitation has been observed to be characterized by a certain type: sometimes there is great vomiting and purging, at other times, one or even both of these symptoms have been entirely wanting; spasms have, in certain instances, been unusually severe; very frequently however, and especially among the natives, they have not occurred at all; great pain and burning at the epigastrium have been constantly observed by some practitioners, while others have found them to occur but seldom. It is evident, therefore, that a description of the disease, which had been drawn from a single visitation, would, in all probability, be quite inapplicable to many of its varieties, and that, in order to arrive at accuracy, we must study the numerous features it has assumed at different times and places. By analysing its phenomena from a multitude of examples, we shall be able to determine their simplest form, and (as it were) the essence of the disease, thus employing a similar method to that which we would adopt in any other question in physics.

The epidemic has sometimes been confounded with common cholera morbus; but it will be hereafter shewn that these are very distinct diseases, although they have been sometimes met with in the same visitations, and even combined together in the same case, thus giving rise to a mixed form of the disease. Different names have been applied to them, but the correct appellations would be catarrhal cholera for the former, and inflammatory cholera for the latter, (*cholera catarrhalis* and *cholera pyretica*:) the reason for which can only be understood after we have studied their symptoms and pathology.

I will first give a simple list of all the symptoms of the epidemic, and will afterwards make observations on each of them, describing their varieties and relative importance, and shall thus be enabled to present a detailed account of all the forms of the malady. I have attempted to place the symptoms, as nearly as possible, in the order in which they usually succeed each other; but many of them being often synchronous, and the disease being sudden in its attack, and rapid in its progress, a mere approximation is here all that can be expected; perfect accuracy, in such circumstances, being out of the question.

1. *Premonitory symptoms.* General uneasiness, lassitude, depression of spirit, and anxiety; simple looseness of the bowels. 2. Vomiting and purging of a sero-fibrinous fluid. 3. A subduing feeling of exhaustion and sinking; jactitation. 4. A sense of uneasiness, burning, and pain at the epigastrium and in the belly. 5. Spasms. 6. Pulse small and accelerated, and at length ceases in all the external parts. 7. Skin becomes gradually colder, and is sometimes covered with a profuse cold sweat, or clammy moisture. 8. The whole surface of the body appears collapsed, the lips become blue; the nails present a similar tint; and the skin of the feet and of the hands becomes much corrugated, and exhibits a sodden appearance; the features of the face collapse; the eyes sink in their orbits, and are sometimes covered with a tenaceous film; the corneæ become flaccid, and the whole countenance assumes a cadaverous aspect strikingly characteristic of the disease. 9. Thirst. 10. Tongue moist, whitish, and cold. 11. No urine or saliva. 12. Voice feeble, hollow and unnatural. 13. Occasionally deafness, and tinnitus aurium. 14. Respiration slow, sometimes oppressed, and breath cold. 15. If blood be drawn, it is dark-coloured, or nearly black, thick, ropy, and in many instances does not separate into serum and crassamentum.

It is worthy of remark, that the disease has been found generally to take place at night, or early in the morning, and death usually occurs within eighteen or twenty hours after the commencement of the attack, and often in a much shorter time.

It is seldom that all the symptoms mentioned in the preceding list have been present in one case; many of them are generally wanting; and cases have sometimes occurred which have proved fatal in a very short period, and in which the only symptoms were anxiety, a subduing sense of sinking, exhaustion, and death. Such, however, are rare, and few cases can be brought forward in which neither vomiting nor purging was present.

1. Although the attack is often very sudden, and without any premonitory symptoms, yet it has generally been observed to be preceded by some of the following: anxiety, with a general uneasiness and depression of spirits, nausea, griping and looseness of the bowels, a sense of debility, and coldness of the skin. No one has studied the premonitory symptoms of the disease so closely as Mr. ANNESLY; and I hope, therefore, that no excuse will be considered necessary for quoting his observations on this important subject, in his own words. He says, "A practitioner possessed of true professional tact will discover in the countenance of the patient the earliest changes which mark the approaching invasion of cholera. The features are expressive of something approaching a state of anxiety, although the patient himself may not be aware of his state, or even that he is at all ailing. If the medical attendant inquire how he feels at this time, he generally answers, "very well;" but, if pressed upon the subject, he acknowledges that he experiences feelings which he cannot distinctly describe, though he feels neither pain nor sickness. His spirits are however low, and there is sometimes a clammy moisture on the skin, and the pulse, though occasionally full and strong, is evidently oppressed and labouring. It is not, however, that kind of pulse which will attract particular attention, unless we are upon the alert for this disease; but, being prepared for such a visitation, it is impossible to mistake it." However much we may be inclined to value the slightest knowledge of any symptoms which might enable us to detect the approach of this dreadful malady, it must at the same time be confessed that the preceding, copied from Mr. Annesly's work, are so obscure and indefinite, that they may easily elude the most vigilant observer. But they are often combined with others which cannot be mistaken; for "during this stage, Mr. Annesly adds, "the patient feels considerable nausea, and has his bowels more freely moved than usual; but the stools then generally consist of such matters as have been lodged in the large intestines, and consequently they present various appearances, according to the state of the digestive organs at the time of invasion. The patient, however, often complains of no actual pain, even on pressure made upon the abdomen, either in this or in the subsequent stage, but what is the result of spasm in the latter. He feels chiefly a great degree of exhaustion, and inability to make the least exertion. Colicky pains are frequently felt in the belly, but they often pass off, or are relieved by pressure, and the free evacuations which take place in this stage. The urine, in the period of invasion, is often in small quantity, and seldom voided. In this stage of the disease, and indeed through its whole progress, in many cases, the abdomen is more than usually tumid,* evidently from congestion of the viscera lodged in this cavity.

A very common mode in which the disease commences is by a simple looseness, or diarrhœa, unaccompanied by spasms or pain, which continues for some hours, or even for a day or two, before any of the more violent symptoms develop themselves.†

The attack is sometimes so very rapid that no premonitory symptoms can be observed, and cases have been recorded in which a patient has been seized instantaneously when going about his usual avocations, apparently in a perfect state of

* But, so far from this being a common symptom, we find it stated in the Report of the Madras Medical Board, that "the abdomen has sometimes been observed to be tumid, but more frequently drawn towards the spine."

† Numerous cases of this description are mentioned in the Madras Report, and many have fallen under my own observation.

health. Mr. Scot supposes that the disease is always of sudden invasion. Mr. Orton says, "the attack of cholera is usually sudden and violent, but, in a great majority of instances, not without some premonitory symptoms;" while Mr. Annesly, on the other hand, asserts that "there are premonitory symptoms, and these of a pathognomonic kind," and that the appearance of a sudden invasion, in some cases, has arisen merely from the patient having not been seen till towards the second stage, when the disease had begun to be formed. I have had occasion, repeatedly, in my own practice, to observe the symptoms which I have mentioned as being indicative of an approach of the disease; but, while I agree so far with Mr. Annesly, I conceive that there can be no doubt of the disease being, in many cases, very sudden in its attack; for my own experience not only affords proofs of this, but numerous others might be adduced in support of it, from the reports of the medical officers of all the three Residencies in India.

2. It often happens that the disease commences at once with a sudden evacuation of all the contents of the bowels, by purging, followed by a repeated discharge, at very short intervals, of a peculiar watery fluid, which in many cases escapes involuntarily on any change of position. There is seldom much griping in this variety, although the calls are frequent and irresistible: the dejections, in general, flow without difficulty or any effort, but sometimes they are thrown out with great force, as if from a syringe. There is sometimes no purging, and the absence of this symptom is supposed by Mr. Scot to denote a peculiar malignancy of the attack.

Vomiting, although a common, is by no means an invariable, symptom of cholera. I have seen cases in which it was entirely absent, and it often occurs only at the commencement of the attack, but soon ceases; the stomach then receiving, and retaining, whatever is poured into it, as if it were a dead substance. The cases in which vomiting has been most urgent have always been found to be less malignant than those in which it was slight, or altogether wanting.

After the first evacuations of the stomach and bowels, of the usual contents, the discharges always consist entirely of a peculiar fluid, which has been very appropriately named the cholera secretion, and is very characteristic of the disease. Although it varies sometimes in its external characters, it has always (as we shall more particularly point out hereafter,) the same chemical composition, consisting of serum and fibrin. In general the fibrin floats in the transparent serum in the form of flakes, or is equally diffused through it, when the whole has a whitish colour, and resembles rice-water.* Sometimes the fibrin occurs in a larger proportion, is intimately mixed with the serum, and forms a variety of the fluid which has somewhat the appearance of chyle, milk, or pus.

In many cases, the cholera secretion, under one or more of the forms just described, is evacuated in prodigious quantity; and "were it uniform," says Mr. Scot, "it might afford us an easy solution of the debility, thirst, thickness of the blood, and other symptoms; but it is unquestionable that the most fatal and rapid cases are by no means those which are distinguished by excessive discharges. We have innumerable instances, on the contrary, of death ensuing after one or two watery stools, without the development of any other symptom affecting the natural functions. Even collapse has come on before any evacuation, by stool, had taken place." It is worthy of remark, however, that the absence of vomiting and purging, in such cases, is not owing to the total absence of the morbid secretion in the alimentary canal, but entirely to the inability of the stomach and intestines to throw it off. I will shew, when treating of the ratio symptomatum of the disease, that, in those cases in which there was no vomiting or purging, the stomach and intestines were found, after death, to be loaded with the cholera fluid; and, had post-mortem examinations been always made in such cases, I am sure that no one would have expressed any surprise at their malignancy; for it is probable that the secretion would have been found even more abundant in them than in ordinary cases. I wish it to be understood, then, that a fluid consisting of serum and fibrin is secreted by the mucous membrane of the stomach and bowels, in all cases of cholera, and is either discharged by vomiting and stool, or retained in the alimentary canal, till the fatal termination of the disease.

The evacuations have been frequently observed to be turbid, and to have a grey, green, or yellow colour; but these appearances can be always traced to the admixture of calomel and other medicines, given for the cure of the disease, and are accordingly never observed at the commencement, but only after the attack has made some progress, or when it has begun to yield. Mr. Annesly has shewn, by experiment, that calomel produces a grey colour when mixed with the secretion, and that, when bile is also added, a green colour is the result.

* This has given rise to the vulgar term for the secretion in India, viz. "conjee evacuations;" conjee being the Hindoostanee word for rice-water; and so well aware is every one of the characteristic nature of this fluid, that, whenever the evacuations of a patient are observed to consist of it, no doubt any longer remains of the disease being cholera.

Blood is sometimes mixed with the evacuations; but this is of rare occurrence, and is probably to be attributed to the rupture of small blood-vessels, occasioned by the great commotion in the alimentary canal. The preceding description, therefore, may be considered as universally applicable to the cholera secretion, except in a few cases, in which its external characters are altered by extraneous circumstances. The evacuations are always without any admixture of bile, the reappearance of which is considered a certain symptom of returning health.

3. One of the most distressing symptoms of cholera is a subduing sense of exhaustion and sinking, which often comes on at the very commencement of the attack, immediately after the first evacuations. The patient is generally overwhelmed with great depression of spirits, is listless, impatient of disturbance, and averse to speak; but it is very remarkable that his mind still remains clear, and he retains the power of expressing his thoughts, almost to the last moment of his existence. Jactitation, which occasionally occurs, and has been observed more frequently in Europeans than in the natives of India, is always to be regarded as a most alarming symptom.

4. Burning and pain at the epigastrium have been very common symptoms in some visitations of cholera, and Mr. Annesly tells us that he never saw a case of the epidemic in which they did not exist. In the cases which have come under my own observation, however, they have, I think, been more frequently absent than present; and in the Report published by the Madras Medical Board, we are told that not only in individuals, but also in epidemic invasions, these symptoms have been altogether wanting. When present, they are often accompanied with pain at the umbilicus, and are generally connected with thirst. In many cases, I have observed that this burning sensation and thirst have not occurred till after the disease has continued for sometime; and I have been inclined, in such cases, to attribute them to the active stimulating medicines which had been poured into the patient's stomach. But this explanation will by no means apply to all cases; for these symptoms frequently make their appearance at the very commencement of the attack, and are then probably owing (as I shall more particularly explain hereafter) to spasms in the stomach and bowels, or to inflammation. The agonies which these symptoms occasion are sometimes most excruciating; but this is seldom the case in the most dangerous form of the disease.

5. Spasms were of such general occurrence in the first visitations of cholera, in 1817 and 1818, as to confer on it the specific name "spasmodic;" but since then, in a great proportion of cases in India, they have been completely wanting. They are generally present in those cases in which there is active vomiting and purging, and appearance of irritation in the alimentary canal, and pain and burning at the præcordium; and they occur more frequently in the robust than in the weak and debilitated, and in European than in native patients.

All the voluntary muscles are liable to be affected by the spasms, but those of the legs and feet are most obnoxious to them. They often extend to the muscles of the abdomen and chest, but seldom attack those of the back, loins, or face. They are sometimes of a tonic, sometimes of a clonic character, and are generally attended with very great pain. Even when there are no spasms or actual pain, the patient is often very restless, tossing about, and appearing to suffer great agony.

Cases accompanied by violent spasms are by no means the most dangerous; and it has been very truly remarked by Mr. Scot that the worst variety of the disease "is that which is noted for the very slight commotion in the system, in which there is no vomiting, hardly any purging, perhaps one or two loose stools, no perceptible spasm, no pain of any kind; a mortal coldness, with arrest of the circulation, comes on from the beginning, and the patient dies without a struggle."

6, 7, 8. None of the symptoms of the epidemic cholera are so truly characteristic and invariable as those which depend upon the retreat of the blood from the surface of the body, including smallness or loss of pulse, collapse, and coldness of the skin. However much they may vary in degree, or in the time of their appearance, they are never wanting in the advanced stage of the disease; and it is only in such cases as are cut short by remedial measures being soon adopted, and consequently in which these symptoms have not time to develope themselves, that they do not occur at all.

The pulse is generally small and accelerated from the commencement; and as the disease advances it gradually diminishes in size, and at length ceases in all the external parts. Sometimes the diminution or loss of pulse is rapid, especially after sudden and copious evacuations of the cholera secretion, by vomiting and stool. On the other hand, it sometimes happens that it is full and strong at the commencement, and diminishes only after the disease has made some progress. Such cases are most frequent among strong plethoric Europeans; and indeed have been seldom observed in the natives of India, owing probably to their vegetable diet, and their abstinence from fermented liquors, rendering their frames less prone to irritation. It is very remarkable that the pulse is often imperceptible for hours before the death of the patient, while at the same time his mind continues clear, and his voluntary muscles obedient to his will: and "instances are not wanting," says Mr. Scot, "of patients being able to walk, and to perform many of their usual avocations, even after the circulation has been so much arrested that the pulse has not been discernible at the wrist: the cases here alluded to are those chiefly in which it has begun by an insidious watery purging; and many lives have been lost in consequence of the patients, under these fallacious appearances, not taking timely alarm, and applying for medical aid."

A species of cholera, known by the name of cholera morbus, differs completely in its symptoms from the usual cases of the epidemic; being characterized by a full strong pulse, severe spasms, pain at the præcordium and in the bowels, and bilious vomiting and purging. It is a much less dangerous disease than the epidemic, is common in other countries as well as in India, and is probably owing to inflammation of some part of the gastro-enteric mucous membrane. Although it shall be more particularly described hereafter, I have thought it necessary to notice it also at present; for it has been observed to prevail during some visitations of the epidemic; and cases have often occurred in which the symptoms of the two diseases have been combined; the attack having commenced with a full strong pulse, hot skin, and active vomiting and purging of bilious matter, and afterwards degenerated into the more dangerous form, accompanied by loss of pulse, collapse, and sero-fibrinous evacuations. It is of much importance that these symptoms should be carefully attended to, since they require alteration in our plan of treatment.

Nothing in this disease arrests the attention of the bystander so forcibly as the sudden change which takes place in the external appearance of the patient's body. The blood being withdrawn from the surface, occasions a collapse or contraction of all the soft parts; the skin of the extremities becomes shrivelled; the hands have a sodden appearance, as if they had been long soaked in water; the bones in different parts of the body project much more than usual; the features of the face shrink, and their expression is completely altered. Often have persons been horror-struck at seeing their friends, whom they had left probably not more than an hour or two, so altered that they could scarcely be recognized, with death marked in their face in the most fearful characters, the cheeks sunk, the lips shrunk and bloodless, the nostrils contracted, the cheek-bones projecting, the eyes sunk in their orbits, with their surface glossy and dimmed, and the brow white and covered with a cold dew. The whole body is at the same time deadly cold, yet the patient complains of feeling hot, and is unwilling to be covered with the bedclothes. Cases have been recorded in which there was no diminution in the heat of the skin, and it has sometimes happened that an increase of temperature has taken place just before death. But it has then been confined to the head and trunk, and has been almost always found to be a fatal symptom, being unconnected with any improvement in the circulation, or in the functions of the lungs. In general, slight coldness of the surface occurs at the very commencement, and gradually increases as the disease advances. Sometimes it is not developed until the malady has made some progress; and cases have not been uncommon in which the attack has been ushered in with a hot skin, and increased action of the circulation. But when the disease has not proved very suddenly fatal, or has been quickly arrested by medicines, this symptom has almost always occurred in the advanced stage.

The state of the perspiration is more variable than that of the temperature of the body. In many cases it is profuse and watery; sometimes it is scanty, and of a clammy nature; it is often confined to the face or brow; and cases have occurred in which it has been entirely wanting. It is always cold, and I have sometimes observed it to have a peculiar cadaverous smell.

9, 10. Thirst is not a very common symptom at the commencement of the disease, except in those cases in which there is pain and burning at the præcordium, and other symptoms of inflammation. Its occurrence in the advanced stage of the common cases of the epidemic have been supposed to be owing (not without reason, I think,) to the large quantity of stimulating medicines which are usually administered. It is often present when the mouth at the same time is moist and cold.

11. The moisture of the mouth in this disease does not appear to proceed from saliva, (the secretion of which is arrested,) but to the peculiar fluid which is thrown out by the mucous membrane of the mouth, in common with all the other mucous membranes of the body. No urine is secreted during the continuance of the disease, no bile, and in fact no fluid, except the cholera secretion. The urinary bladder is often found to contain a fluid secreted by its lining membrane, and similar to that expelled from the alimentary canal.

The reappearance of urine, or bile, is always considered as a decided indication of returning health.

12. In the advanced stage of the disease the voice becomes altered, hollow, feeble, and unnatural; but it is surprising how distinctly the patient is able, in many cases, to articulate until within a few minutes of his end, and long after his pulse has become imperceptible.

13. Deafness, and tinnitus aurium, are mentioned by some authors as of very frequent occurrence, but I scarcely ever observed them in my own practice. They are probably present only when there is much congestion in the vessels of the brain. Vertigo is not uncommon, and sometimes occurs at the very moment of seizure, in rapid cases of the disease. Coma has been mentioned as not an unusual symptom. It may have occasionally occurred towards the fatal termination, but it is certainly not common; for, although the patient is generally in a dozing state, with slow breathing, and is very unwilling to be disturbed, yet, when roused, his mental faculties are quite collected, and he answers distinctly any questions put to him.

14. The respiration is generally slow, sometimes oppressed and irregular, occasioning great distress to the patient. The breath is usually cold; and it would appear, from the experiments of Dr. Davy, that it contains less carbonic acid than in a state of health. In cases where there is no spasm, the mechanical part of respiration continues to be performed to the last, only becoming gradually slower.

15. The state of the blood in cholera has universally excited the utmost attention. In well-marked cases of the epidemic, it may be said to be invariably of a dark colour, in the arteries as well as veins; and, next to the peculiar secretion from the mucous membranes, this is probably one of the most constant symptoms of the disease. But it is not at the very commencement of an attack that this change in the colour of the blood is observed, and it does not arrive at its greatest intensity until the disease has made some progress. Besides having a dark, and in many instances an almost black colour, it also undergoes other important changes: sometimes it is thick, like tar, sometimes thin and watery; it always flows with difficulty, and seldom separates into serum and crassamentum, remaining in a perfectly fluid state, or acquiring the consistence of liquid honey, or of tar. Although these changes are less in some cases than in others, yet I believe the blood is invariably darker coloured than in health. This being also observed, however, to take place in various other diseases in India, such as certain cases of fever, rheumatism, &c., it will not alone serve as a diagnostic symptom of cholera. Mr. Scot says, "The blood is generally found to be less changed in appearance in those cases of cholera which have been ushered in with symptoms of excitement, than where the collapsed state of the system has occurred at an early period." I have observed in my own practice, and it has also been often observed by others, that, upon venesection, the blood very often becomes lighter coloured, and more healthy in its appearance, as it continues to flow, accompanied at the same time with an evident amendment in the general state of the patient.

Such then are the symptoms of epidemic cholera, a disease which, if left to take its course, is generally fatal; nay, in many cases, if proper remedies be delayed even an hour or two, the patient's fate is sealed. More rapid in its progress than plague, often more cruel in the tortures it inflicts upon its victims, it has in many countries, when not arrested by the skill of the physician, cut off thousands and tens of thousands, in a fearfully-short time. But it is consoling to know that medicine has the power, in a large proportion of cases, to subdue it; and perhaps never has the triumph of the medical art over disease been so conspicuous as in epidemic cholera; it may be said, with truth, that it has plucked thousands from the jaws of death, for death is the ordinary termination of this dreadful malady, when not counteracted by art.

It has been already mentioned that cases of cholera morbus have sometimes accompanied visitations of the epidemic, and that the two have been occasionally conjoined; thus producing modified forms of the disease, which will now require to be described.

Cholera morbus is a well-known disease in all countries: its symptoms are—pain and burning in the stomach and bowels; pulse strong and full; bilious vomiting and purging; spasms; hot and dry skin; great thirst; anxious, distressed, and flushed countenance. The efforts to vomit are strong, irresistible, and painful, but they produce only scanty evacuations, which consist almost entirely of bile; the purging is also painful, and the stools contain nothing but bile, with sometimes a little blood. The pain in the stomach and intestines is generally excruciating, and is accompanied by severe spasms, which also attack the muscles of the legs, and sometimes almost all the voluntary muscles of the body. The thirst is constant and burning, and is not alleviated by any kind of drink, which is immediately rejected by the stomach. The countenance is flushed, expressive of great pain, and the eyes are generally inflamed. The blood flows freely from a vein, is not darker than usual, and separates readily into serum and crassamentum. Returning health is indicated by the pulse becoming soft, by perspiration bursting out upon the skin, and by the evacuations becoming more copious, less bilious, and expelled with ease, and without pain. All these symptoms are exactly the reverse of those I have already described as belonging to the epidemic cholera.

The combination of the two diseases may give rise to two principal modifications: first, the attack may be ushered in under the form of cholera morbus, and may degenerate into that of the epidemic; secondly, the symptoms of the two may be combined throughout the case, and thus modify each other. Both have been noticed by different authors who have treated of cholera; but having been entirely overlooked by others, we may conclude that they have not been of very frequent occurrence.

The first modification is described in Mr. Orton's work on Cholera, in the following words: "I have found a very considerable number of cases," says he, "exhibiting singly, or in partial combination, every possible degree, and almost every kind, of increased action. Spasms, such as not to fall short of the highest degree of tetanus; retching, and spasms of the intestines equalling colica pictonum; very full, hard, and quick pulse; hot skin and flushed surface; evacuations of bile, both by vomiting and stool, from the commencement of the attack. And, finally, I have seen some of these cases passing into the low form of the disease; the circulation passing suddenly from extreme strength to extreme weakness; the skin, from being hot and dry, becoming deadly cold, and bathed in sweat; and the increased flow of bile succeeded by white stools and vomiting, clearly indicating the total suppression of that secretion." Nothing can be more distinct than this description; in the first part of which we find the most marked features of cholera morbus, and in the latter part the no-less characteristic symptoms of the epidemic; and it could not have been stated in clearer terms, even had it been given for the express purpose of illustrating the views I have taken of the disease. In some cases the symptoms of the cholera morbus continue but a very short time; a few bilious evacuations, and a quick hard pulse, being soon replaced by watery stools, a small pulse, collapse, &c., and the disease becoming thus converted into the usual form of the epidemic.

The symptoms of the second modifications, or of those cases in which the two diseases are blended together, vary considerably, according as the one or the other predominates. If the discharges are not very copious, the pulse generally keeps up, and the skin continues warm; but, as soon as the evacuations become watery, and the true cholera secretion makes its appearance, then the blood retreats from the surface towards the viscera, the skin becomes cold, the pulse small, and collapse takes place. Sometimes the pulse has continued pretty full, and the skin hot, when there has been no bile in the evacuations. Sometimes the evacuations are more abundant than in common cases of cholera morbus, and contain a good deal of serum, but are at the same time mixed with bile; the heat of the skin suffers little diminution, and the pulse is variously affected, being occasionally quicker and smaller than usual, and in other instances continuing soft and full. Cases, again, have been met with in which there was no vomiting, but constant purging, and in which the skin continued hot, and the pulse but little affected. It would be endless, however, to mention all the varieties which have been observed in different visitations of the disease.

All these mixed cases are less dangerous, and less rapid in their progress, than the pure catarrhal cholera, and are much more frequent among the robust and plethoric than among the weak, and, consequently, among Europeans than among natives of India.

Dr. Monat describes* a visitation of cholera which occurred in his Majesty's 14th regiment, at Berhampore, in March and April 1828, which affords a good example of the manner in which the inflammatory and catarrhal cholera are sometimes blended. Many of the cases were of the pure catarrhal form, the attack having been sudden, the evacuations copious and without bile, the pulse small or imperceptible, and the skin cold and shrunk. There appears to have been no unalloyed case of the pure inflammatory cholera, but numerous cases partook of many of the characters of that disease, some or all of the following symptoms having been present: pain at the præcordium, or in the belly, purging and vomiting of bilious matter, skin hot, pulse quick; and these were either accompanied or followed, unless the disease was soon checked by medicine, by the more characteristic features of the catarrhal cholera. The latter was by far the more fatal form of the disease; "but," says Dr. Monat, "several with bilious vomiting terminated fatally."

It is to be regretted that the author has given a very im-

* Transactions of the Medical and Physical Society of Calcutta, vol. iv.

perfect description of the post-mortem appearances, and he has given none of his cases in detail; but I have selected a few from a short abstract at the end of his memoir, and have inserted them in the appendix, in order to shew, as far as possible, the nature of the symptoms by which these mixed cases were characterised.

Cases have sometimes occurred in which the catarrhal cholera has been followed by inflammation, and at length by ulceration, or other organic lesions of the bowels; but which have been very rare, having not been even mentioned by the greater number of writers, and I know no author who has described them in detail except Mr. Jameson. I will therefore give that author's description in his own words.

"When the disease," he says, "ran its full course with Europeans, and with natives of robust athletic make, the following appearances generally presented themselves. What may be termed the cold stage, or state of collapse, usually lasted from twenty-four to forty-eight hours, and was seldom of more than three complete days' duration. Throughout the first twenty-four hours, nearly all the symptoms of deadly oppression, the cold skin, and oozing of clammy sweat from every pore, the feeble pulse, occasional vomiting, purging, and cramps, the thirst, and anguish, continued undiminished. Then the system shewed symptoms of revival, the vital powers began to rally, the circulation and heat to be restored, and the spasms, sickness, and desire to go to stool, to be considerably lessened. The warmth gradually returned, the pulse rose in strength and fullness, and then became sharp, and sometimes hard; the tongue got more deeply furred; the thirst continued, with less nausea; the stools were no longer like gruel, or rice-water; they usually, between the third and sixth day, became first brown and watery, then dark-green, black, and pitchy, and the bowels, during many days, continued to discharge immense loads of vitiated bile, until, with returning health, the secretions of the liver and other viscera gradually put on a natural appearance. These discharges were generally hot and acrid, and passed with griping and tenesmus. Sometimes they were of a bright-yellow colour, and the surcharge of bile was so great as to be ejected in a pure stream from the stomach. It was remarked that, when the motions consisted of a chocolate-coloured fluid, with flocculi swimming in it, the patient rarely recovered.

"The fever, which almost invariably attended this second stage of the disease, may be considered to have been rather the result of an effort of nature to recover herself from the rude shock which she had sustained, than as forming any integrant and necessary part of the disorder itself. It partook much of the nature of the common bilious attacks of these latitudes. There was the hot dry skin; the foul, deeply-furred, dry, tongue; parched mouth; thirst; sick stomach; depraved secretions; restlessness; watchfulness; and quick, variable pulse; sometimes with delirium, stupor, and other marked affections of the brain.

"Generally, when the disorder proved fatal, after reaching this stage, the tongue, from being cream-coloured, got brown, and sometimes black, hard, and more deeply furred; the teeth and lips were covered with sordes; the state of the skin varied, chills alternating with heats; the pulse became extremely quick, weak and tremulous; hiccough, catching of the breath, great restlessness, and deep moaning, succeeded; and the patient soon sunk, incoherent and insensible, under the debilitating effects of low nervous fever, and frequent dark, tarry, alvine, discharges.

"In other cases, this secondary period ran a somewhat different course. As the action of the heart and arteries was renewed, and the natural warmth of the body returned, an unusual degree of energy succeeded. The brain was evidently affected, and the patient was quite insensible to the great danger into which he had fallen. The pulse rose as high as 120; great heat, especially over the large cavities, was complained of. There was extreme agitation and distressing thirst. The patient continually called for cold water, to relieve the burning sensation of the abdomen. Sometimes a warm perspiration broke out near the wrists and forehead, which afforded temporary relief to his sufferings. To this state of excitement, that of collapse quickly succeeded. There was then great prostration of strength, the bowels became quite torpid; severe pains occurred low down in the abdomen, near the site of the rectum, which were always aggravated upon stools being procured by medicine. The state of the stomach now excited surprise; its natural irritability was entirely gone, and the most nauseating medicine could be poured into it without exciting vomiting. It rarely occurred that the patient survived the great sinking produced by this stage; and even when good fortune, and the strength of his constitution, carried him through it, he suffered long after from debility and disordered bowels."*

I will here recapitulate some of the most important facts detailed in this chapter, in order to bring them at once plainly before the reader. 1. There are two distinct kinds or spe-

* Bengal Report, pp. 53—56.

cies of cholera, viz. the catarrhal and inflammatory (cholera catarrhalis, and cholera pyretica.) The most constant and characteristic symptoms of the former are copious discharges by vomiting and stool of a sero-fibrinous fluid; retreat of the circulating fluid from the surface towards the viscera; dark coloured blood; collapse; cold damp skin. The symptoms of the latter are vomiting and purging of bile; pulse full and hard; skin hot and dry; spasms. 2. The catarrhal cholera is the most common form of the disease in epidemic visitations, and is much more dangerous than the other. 3. The inflammatory sometimes degenerates into the catarrhal cholera; and the two are occasionally combined in the same case.

*Prognosis.*

Little need be said under this head, after the numerous observations I have already made upon the nature of the different symptoms. When an attack commences with symptoms of inflammatory cholera, or with increased action of the circulation, and the patient is seen before the violent symptoms of this form of the disease, such as severe vomiting, pain, heat of skin, and spasms, give way to collapse, the case may be considered completely within the reach of art; but when these symptoms begin to pass off, and to be followed by cold skin, shrunk features, loss of pulse, watery purging, &c., the case must be considered as one of great danger, and requiring the most prompt measures. In the true catarrhal cholera every moment is of importance, and the shortest delay may seal the fate of the patient; for if he be seen before the blood has left the surface to accumulate about the internal viscera, before the pulse has ceased at the wrist, and the skin has become cold, we may entertain great hopes of being able to overcome the malady. But if the pulse has ceased, the skin become cold, the features shrunk, and the vomiting and purging have stopped, then our prognosis must be bad indeed.* Even such cases, however, must not be abandoned; I have seen many of them brought round by energetic measures. It must be evident, from all that has been said, that the diagnosis, in cholera, must be principally derived from the pulse. If we find it to increase in strength and size, there is cause for hope; and if this favorable symptom be accompanied by the resto-

* Mr. Scot says, "The danger of cholera may thus be said to be manifested, not by the violence of morbid actions, but by the diminution or cessation of natural actions." This however is only apparent; for, if we look below the surface, we find that the danger may be measured by the violence of that morbid action which we have shewn to constitute the disease, viz. the catarrh of the mucous membranes, while most of the other natural functions are diminished.

ration of warmth on the surface, and the reappearance of the secretion of urine and bile, the patient may be considered safe, although still requiring the most watchful care to prevent a relapse; for a draught of cold water, or exposure to external cold, have sometimes been known to cause a renewal of the disease, after the patient had been considered out of danger.

